# Effectiveness of a Covid mRNA Vaccine Against Two Early Variants: A Reanalysis of Published Data

**DOI:** 10.7759/cureus.90666

**Published:** 2025-08-21

**Authors:** Eyal Shahar

**Affiliations:** 1 Department of Epidemiology and Biostatistics, Mel and Enid Zuckerman College of Public Health, University of Arizona, Tucson, USA

**Keywords:** covid, effectiveness, healthy vaccinee bias, immortal time bias, vaccine

## Abstract

Introduction: Two strong biases, immortal time and healthy vaccinee, have not received the attention they deserve in observational studies of Covid vaccines. The former arises from excluding early events in the vaccinated, and the latter from residual confounding by health-related attributes. Both biases lead to overestimation of vaccine effectiveness.

Methods: Published data from a test-negative case-control study of an mRNA vaccine were reanalyzed. The original study estimated the effect on infection by two early variants, Alpha and Beta, and the effect on a severe outcome (severe, critical, or fatal infection) by either. Immortal time bias was removed by considering all events and estimating built-up immunity by 27 days after the first dose and by 13 days after the second. Healthy vaccinee bias was reduced by applying a bias factor correction derived from the pseudo-effect on non-Covid death (negative controls).

Results: All estimates were strikingly different from the original results. Effectiveness against infection by either variant might have been negative in the first two weeks after the first dose and has reached only 41% and 15% against Alpha and Beta, respectively, by the end of the fourth week. At the time of full immunity, it was around 60% against Alpha and below 50% against Beta, not over 95% as originally computed for each variant. Accounting for immortal time bias alone, effectiveness against a severe outcome changed from 100% to about 50%. Then, a rudimentary correction for healthy vaccinee bias not only eliminated meaningful benefit but also raised the possibility of negative effectiveness in the first month or so.

Conclusion: Immortal time bias and healthy vaccinee bias have severely distorted estimates of Covid vaccine effectiveness. This analysis should be replicated in other observational studies to corroborate or refute the findings.

## Introduction

Two strong biases have threatened the validity of observational studies of Covid vaccines: immortal time bias and healthy vaccinee bias [1-3]. So far, neither has received the attention it deserves.

Immortal time bias arises from ignoring events that have occurred during the first 14 days after the first dose, an analytical decision that has been adopted by most researchers. Formally called case-counting window bias [4], the exclusion of early events leads to a special kind of information bias [5], by analysis. The bias can be detected and avoided if the missing data are disclosed. Unfortunately, almost all publications do not present the crucial data. One exception is the focus of this article.

Unlike immortal time bias, healthy vaccinee bias was studied or acknowledged in numerous publications, but it was not considered analytically. It is a type of confounding bias. People who are vaccinated are healthier on average than people who are not, and therefore, the former are at lower risk of death and severe outcomes [3]. Likewise, those who continue to take another dose are healthier than those who do not [6-8].

Most importantly, the bias is not removed by conventional methods of deconfounding, neither by careful matching nor by various types of modeling [6,8-12]. Sometimes, adjustment hardly changes the confounded estimates [9].

The healthy vaccinee phenomenon seems to be universal, observed wherever it had been studied intentionally or coincidentally, in the USA [9], the UK [10], Canada [11], Norway [12], Sweden [13], Austria [14], the Czech Republic [7], Israel [6], and Qatar [8].

Published data from Qatar were used to reanalyze vaccine effectiveness, accounting for immortal time bias and healthy vaccinee bias.

## Materials and methods

The causal structures

**Figure 1:**
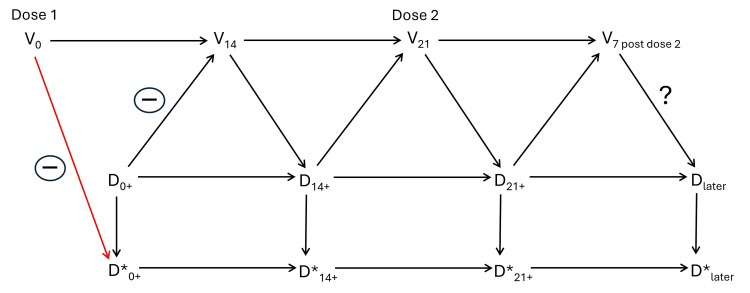
Causal structure of immortal time bias, resulting from excluding deaths of vaccinated people during the first 14 days after the first dose V: vaccination status, D: vital status, D*: measured (classified) version of D, subscripts: time points, plus sign: a time in between, question mark: effect of interest

Figure 1 shows the causal structure of immortal time bias [1], based on a recent, generic description [2]. V is vaccination status, D is vital status, and D* is the measured (classified) version of D. Subscripts indicate time points, and a plus sign denotes a time in between. For example, D_0+_ is a time point between one day and 14 days after the first dose. The question mark denotes the effect of interest, of being fully vaccinated (seven days after the second dose).

Since immunity does not develop immediately, there is no arrow from V_0_ to D_0+_, and the path V_0_-->D_0+_-->D*_0+_ does not exist. The red arrow from V_0_ to D*_0+_ is not natural causation. Rather, it was created by imposing zero frequency of events in the vaccinated, which amounts to a made-up inverse association between V_0_ and D*_0+_ (negative sign). This inverse association is carried forward to D* at later times, because the classification at each time point affects the next classification. (For example, conditional on D_0+_, the probability of D*_14+_=dead is 1 when D*_0+_=dead and not 1 when D*_0+_=alive.)

The path from V_0_ to D*_later_ through intermediary D* variables (bottom of Figure 1) is a source of information bias [5] because this path supplies unwanted information on the value of D*_later_. It was created by the red arrow, the analytical decision to exclude deaths of vaccinated people in the first 14 days. Ironically, the formal justification for that decision (no effect is expected until immunity develops) ends up creating or adding an artificial beneficial effect.

Death may be replaced with severe disease, hospitalization, symptoms, or infection. The causal structure applies to any event.

**Figure 2:**
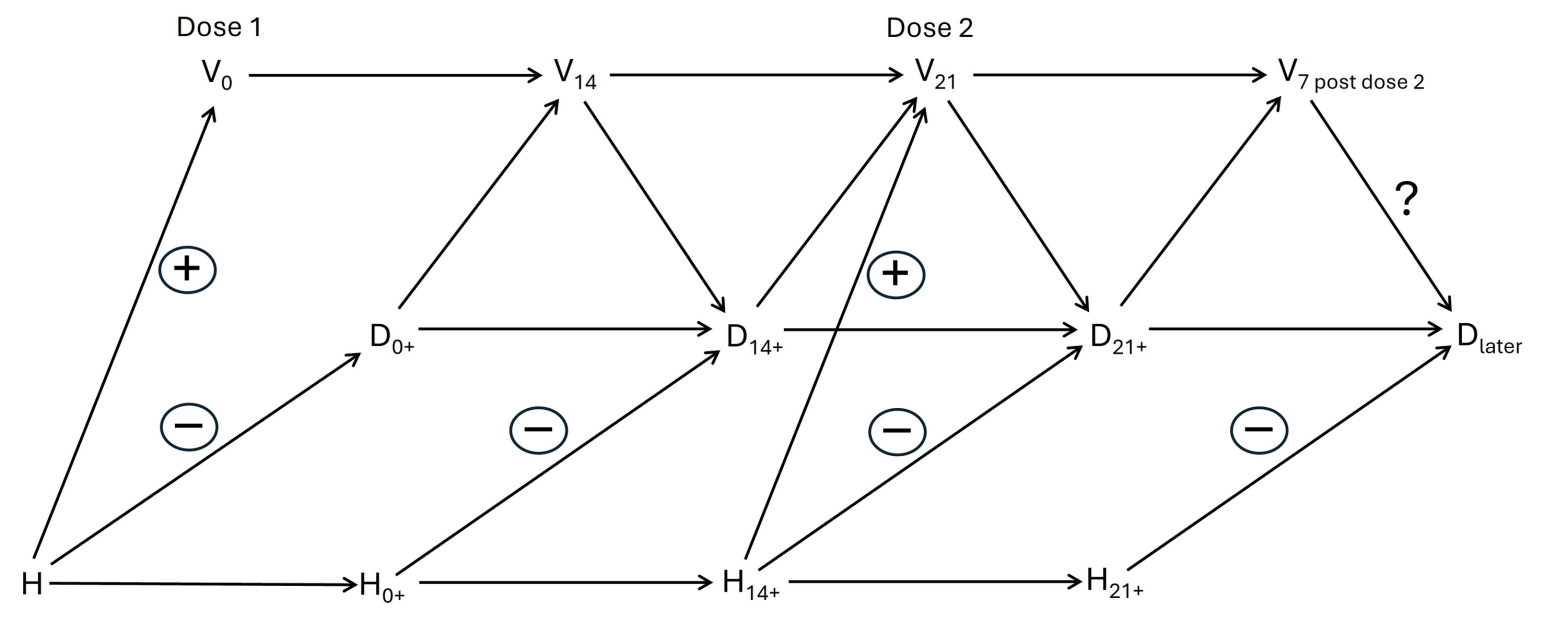
Causal structure of healthy vaccinee bias V: vaccination status, D: vital status, subscripts: time points, plus sign: a time in between, question mark: effect of interest, H: health status

Figure 2 shows the causal structure of healthy vaccinee bias. The variable H (health status) represents unknown confounders that are not accounted for in the design or analysis.

Data

Numerous publications from Qatar have been devoted to various aspects of the Covid pandemic. Although the population of Qatar is relatively young and unique in some respects, it is also unique in crucial aspects of Covid data. Both vaccination status and outcomes have been meticulously ascertained in Qatar, and the publications are exemplary, providing details at a level that is rarely found in the literature.

In July 2021, researchers from Qatar published an article in which they estimated the effectiveness of a Covid mRNA vaccine (Moderna) against two variants that followed the wild-type variants [15]: B.1.1.7 (Alpha) and B.1.351 (Beta). They used the test-negative case-control design, with matching, and focused on three main outcomes: infection by each variant, regardless of symptoms, and a combined outcome of severe, critical, or fatal infection, regardless of variant. The number of deaths was too small for a separate analysis.

In the original paper [15], data analysis followed the usual format. Effectiveness was estimated at various intervals following the first dose, including the first two weeks: 0-6 days, 7-13 days, 14-20 days, and 21-27 days. The second dose of the Moderna vaccine was administered around 27 days after the first, and the authors computed effectiveness in two subsequent intervals: 0-6 days and 7-13 days. Extended data also show cumulative counts over a somewhat longer follow-up.

The estimated effectiveness of full vaccination (7-13 days after the second dose) was over 95% for each outcome (100% against severe, critical, or fatal disease). Like others, the authors did not account for immortal time bias or healthy vaccinee bias. The data was reanalyzed to account for both.

To further elaborate, the source of data that has been used for the detailed analysis is that by Chemaitelly et al. [15]. Moreover, the tables in the Results section clearly show the source data (regular font), whereas the new analysis is shown in bold print.

Analysis

To remove immortal time bias, no events were excluded, and effectiveness was estimated by a given day post-dose 1, rather than at different intervals. The series of consecutive estimates (by day 13, by day 20…, by day 13 post-dose 2) reflects the process of built-up immunity.

This approach not only avoids immortal time bias but also tries to mimic the analysis of a randomized trial, which is focused on the intention-to-treat. Since an observational study should emulate a randomized trial as closely as possible, the initiation of the two-dose protocol (first dose) is the best substitute for an intention-to-treat analysis. Moreover, this approach was recommended in the methodological literature:

“With observational data, the best way to emulate time zero of the target trial is to define time zero to be the time when an eligible individual initiates a treatment strategy” [16].

Likewise, all subsequent events should be counted, as in a randomized trial.

Healthy vaccinee bias cannot be removed by an exact method. However, a first-order correction can be achieved by computing a bias factor, the pseudo-effect on non-Covid death (called “negative controls” [17]). Then, a biased estimate of the effect on Covid death (or on severe Covid) is divided by that number or multiplied by the inverse. The origin of the method and the rationale are explained elsewhere [18].

Based on data from different countries, the multiplier ranges from 2 to 3.5. For example, an estimated risk ratio of 0.4 would be close to the null after correction (0.4*2) or worse (e.g., 0.4*3.5). In another publication from Qatar [8], the hazard ratio of non-Covid death over six months post-vaccination was 0.35 (95% confidence interval (CI): 0.27-0.46). Taking the inverse, the multiplier ranges from 2.2 to 3.7. The inverse of the upper bound of a 99% confidence interval was 2.0. All three numbers were applied in the correction (sensitivity analysis).

## Results

Table 1 shows results for infection by B.1.1.7 (Alpha). The format will be replicated for B.1.351 and severe outcomes.

**Table 1:** Effectiveness of the Moderna mRNA vaccine against infection by B.1.1.7 (Alpha) *Longer follow-up PCR: polymerase chain reaction

	Days	Cases (PCR positive)		Controls (PCR negative)		Odds ratio	Effectiveness
	Post-dose 1	Vaccinated	Unvaccinated	Vaccinated	Unvaccinated		
	0-6	166	23661	170	23657	0.98	2.4%
	7-13	202	23681	187	23696	1.08	-8.1%
	14-20	32	23669	173	23528	0.18	81.6%
	21-27	9	23656	160	23505	0.06	94.4%
Since dose 1	0-27	409	~23667	690	~23597	0.59	41%
	Post-dose 2						
	0-6	4	21454	196	21262	0.02	98.0%
	7-13	1	21377	119	21259	0.01	99.2%
Since dose 1	By day 13	414	~22916	1005	~22818	0.41	59%
Since dose 1*	Extended data in Figure 1	444	24590	1129	23904	0.38	62%

Numbers in bold print were computed. All other numbers were taken from the article. The number of controls over 27 days and until 13 days after dose 2 was computed by taking the average of the counts in consecutive periods. The error should be small because the number of unvaccinated controls was similar to the number of unvaccinated cases in every interval, and a simple ratio of vaccinated cases to vaccinated controls is similar to the odds ratio.

By the end of the fourth week after the initiation of the vaccination protocol, effectiveness against Alpha reached about 40%, far from the biased estimate of nearly 95%. By the time full immunity was developed, effectiveness reached about 60%, not 100%.

The sample size was substantially larger for the Beta variant, and effectiveness was lower: 15% by the end of the fourth week and below 50% when fully immune (Table 2).

**Table 2:** Effectiveness of the Moderna mRNA vaccine against infection by B.1.351 (Beta) *Longer follow-up PCR: polymerase chain reaction

	Days	Cases (PCR positive)		Controls (PCR negative)		Odds ratio	Effectiveness
	Post-dose 1	Vaccinated	Unvaccinated	Vaccinated	Unvaccinated		
	0-6	535	47359	558	47336	0.96	4.2%
	7-13	854	47372	566	47660	1.52	-51.8%
	14-20	270	47329	516	47083	0.52	47.9%
	21-27	114	47249	431	46932	0.26	73.7%
Since dose 1	0-27	1773	~47327	2071	~47253	0.85	15.0%
	Post-dose 2						
	0-6	42	45280	719	44603	0.06	94.2%
	7-13	18	45064	498	44584	0.04	96.4%
Since dose 1	By day 13	1833	~46608	3288	~46366	0.55	45%
Since dose 1*	Extended data in Figure 1	2093	50349	3799	48643	0.53	47%

**Figure 3:**
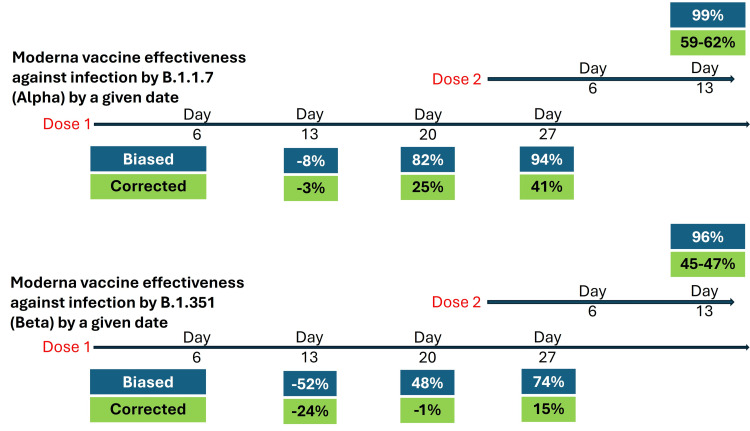
Effectiveness of the Moderna mRNA vaccine against two variants before and after the removal of immortal time bias

Figure 3 compares the results from the article with the computed effectiveness at four time points after the vaccination protocol was initiated.

The numbers in blue cells are estimates of effectiveness in the preceding interval, as reported in the article. The numbers in green cells show built-up effectiveness by a given day after the first dose.

Immortal time bias is observed at each time point. There is evidence of early negative effectiveness, notably against the Beta variant.

Table 3 shows the results for a severe outcome: severe infection, critical infection, or fatal infection. Again, there are striking differences after the removal of the bias.

**Table 3:** Effectiveness of the Moderna mRNA vaccine against severe, critical, or fatal infection *Longer follow-up PCR: polymerase chain reaction

	Days	Cases (PCR positive)		Controls (PCR negative)		Odds ratio	Effectiveness
	Post-dose 1	Vaccinated	Unvaccinated	Vaccinated	Unvaccinated		
	0-6	53	4012	65	4000	0.81	18.7%
	7-13	85	4016	68	4033	1.26	-25.5%
	14-20	18	4007	60	3965	0.3	70.3%
	21-27	4	3997	50	3951	0.08	92.1%
Since dose 1	0-27	160	~4008	243	~3987	0.66	34%
	Post-dose 2						
	0-6	0	3432	62	3370	0	100%
	7-13	0	3399	29	3370	0	100%
Since dose 1	By day 13	160	~3811	334	~3782	0.48	52%
Since dose 1*	Extended data in Figure 3	182	4315	390	4107	0.44	56%

**Figure 4:**
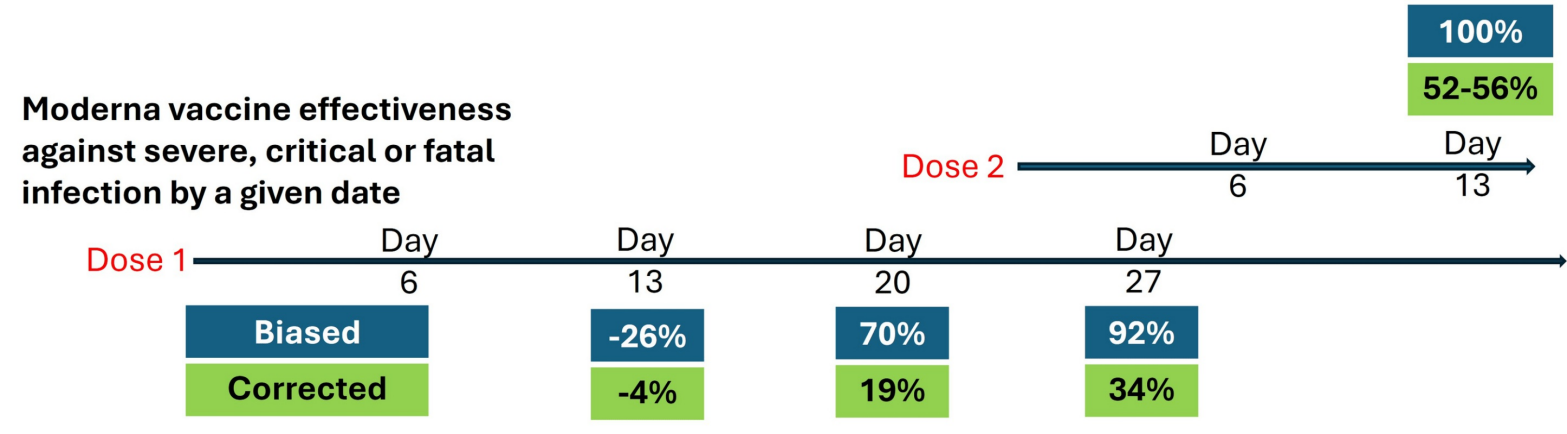
Effectiveness of the Moderna mRNA vaccine against severe outcomes of either variant, before and after the removal of immortal time bias

Figure 4 displays the contrast between biased estimates and corrected estimates.

Removal of immortal time bias has changed the effectiveness of the first dose (by day 27) from 92% to 34%. By two weeks after completing the two-dose protocol, effectiveness reached about 50%, not 100%. Again, there is a hint of negative effectiveness early on.

To account for healthy vaccinee bias, three correction factors were applied to two estimates of effectiveness against severe, critical, or fatal disease (Table 4).

**Table 4:** Effectiveness of the Moderna mRNA vaccine against severe, critical, or fatal infection, corrected for healthy vaccinee bias *Adjusted hazard ratio in the first six months of follow-up (vaccinated versus unvaccinated) in Qatar: 0.35 (95% CI: 0.27-0.46). The upper bound of a 99% CI is 0.50. CI: confidence interval

Hazard ratio of non-Covid death*	Bias factor (1/hazard ratio)	Computed odds ratio (interval)	Corrected odds ratio	Corrected effectiveness
0.27	3.7	0.66 (0-27 days)	2.44	-144%
0.46	2.2		1.45	-45%
0.5	2		1.32	-32%
0.27	3.7	0.44 (extended data)	1.63	-63%
0.46	2.2		0.97	3%
0.5	2		0.88	12%

Following either correction, effectiveness by 27 days after the first dose was negative. The best result after extended follow-up post-dose 2 is near-zero effectiveness. The worst is negative effectiveness again.

## Discussion

No well-designed randomized trial has estimated the effectiveness of a Covid vaccine against severe outcomes [19]. None has tested the mRNA vaccines against infection by new variants. After the original trials, all estimates have been derived from observational studies.

A study from Qatar has estimated the effectiveness of a Covid mRNA vaccine against two variants, Alpha and Beta. A reanalysis of the data showed results that were strikingly different from the original results. After removal of immortal time bias, effectiveness against infection by either variant might have been negative in the first two weeks after the first dose and has reached only 41% and 15% against Alpha and Beta, respectively, by the end of the fourth week. At the time of full immunity, it was around 60% against Alpha and below 50% against Beta, not over 95% as originally computed for each variant. Given the waning of protection, any modest benefit quickly faded within a few months.

Furthermore, the effectiveness of a vaccine against a viral infection is not meaningful unless the vaccine also protects against serious consequences. The original result of 100% effectiveness against severe, critical, or fatal infection did not hold. Accounting for immortal time bias, the effectiveness changed to about 50%. Then, a rudimentary correction for healthy vaccinee bias not only eliminated meaningful benefit but raised the possibility of negative effectiveness.

Is it possible that so many observational studies have been wrong?

One historical example comes to mind. In the 1980s, observational studies consistently reported remarkable effects of hormone replacement therapy on the risk of cardiovascular disease in post-menopausal women. Those promising results were followed by randomized trials, which not only failed to show benefit but suggested harm [20]. Numerous explanations were proposed for the misleading results of observational studies, among which was healthy user bias. Women who started taking replacement hormones might have been healthier than women who did not, and standard methods of adjustment failed to remove the bias. The healthy vaccinee effect is a similar phenomenon.

Discarding early events is a new method to analyze data. It is a major source of bias in an observational study [4] and is unjustifiable even in the context of a randomized trial. Even when “no expected benefit” is an acceptable premise, “no expected harm” is not. By analogy, chemotherapy for acute leukemia is not expected to benefit patients before the end of the induction period. Has any randomized trial of chemotherapy excluded early deaths?

The analytical approach that was used here is easy to replicate in other publications of Covid vaccines because the data is available to their authors. The robustness of these findings should be examined by others, whatever the outcome might be: corroboration or refutation. We must learn from mistakes, if found, because the Covid pandemic is unlikely to be the last pandemic.

## Conclusions

Immortal time bias and healthy vaccinee bias have severely distorted estimates of Covid vaccine effectiveness from observational studies. The former may be removed by considering built-up immunity after various days post the first injection. The latter may be corrected by applying a bias correction factor. Applying both corrections to published data, all estimates of effectiveness were strikingly different from the original results. Effectiveness against infection by two early variants was much lower than 90%, and effectiveness against severe outcomes was at best minimal, if not negative. In accord with the scientific method, this analysis should be replicated by other studies to corroborate or refute the findings.
